# 3D Radiation Therapy or Intensity-Modulated Radiotherapy for Recurrent and Metastatic Cervical Cancer: The Shanghai Cancer Hospital Experience

**DOI:** 10.1371/journal.pone.0040299

**Published:** 2012-06-29

**Authors:** Su-Ping Liu, Xiao Huang, Gui-Hao Ke, Xiao-Wei Huang

**Affiliations:** Department of Gynecological Oncology, Fudan University Shanghai Cancer Center, Shanghai, China; University of Nebraska Medical Center, United States of America

## Abstract

We evaluate the outcomes of irradiation by using three-dimensional radiation therapy (3D-RT) or intensity-modulated radiotherapy (IMRT) for recurrent and metastatic cervical cancer. Between 2007 and 2010, 50 patients with recurrent and metastatic cervical cancer were treated using 3D-RT or IMRT. The median time interval between the initial treatment and the start of irradiation was 12 (6–51) months. Salvage surgery was performed before irradiation in 5 patients, and 38 patients received concurrent chemotherapy. Sixteen patients underwent 3D-RT, and 34 patients received IMRT. Median follow-up for all the patients was 18.3 months. Three-year overall survival and locoregional control were 56.1% and 59.7%, respectively. Three-year progression-free survival and disease-free survival were 65.3% and 64.3%, respectively. Nine patients developed grade 3 leukopenia. Grade 5 acute toxicity was not observed in any of the patients; however, 2 patients developed Grade 3 late toxicity. 3D-RT or IMRT is effective for the treatment of recurrent and metastatic cervical cancer, with the 3-year overall survival of 56.1%, and its complications are acceptable. Long-term follow-up and further studies are needed to confirm the role of 3D-RT or IMRT in the multimodality management of the disease.

## Introduction

Despite improvements in the outcomes of single or combined modality treatment for achieving higher local control of cervical cancer, locoregional recurrences or distant metastasis after initial (surgical or radiation) treatment remain a major therapeutic challenge. A 10–20% recurrence rate has been reported following primary surgery or radiotherapy in women with stage IB–IIA cervical tumors. Moreover, if lymph node metastases are present at diagnosis or if the tumor is in a locally advanced stage, the local recurrence rate increases to ≥70% [1], [2], [3].

Although chemotherapy remains the major treatment modality in the management of patients with recurrent and metastatic cervical cancer, its effectiveness is relative poor comparing to other gynecologic malignancies. Disruption of blood vessels by operation or high doses of radiation may lead to lower perfusion of the relapsed cancer. Although various regimens have been used in a various studies, the response rates are low and the toxicities are severe. Cisplatin has emerged as the most active single agent with overall response rates of 19% [4]. Recent phase III trial has documented response rates of 29.1%, 25.9%, 22.3% and 23.4% when cisplatin has been combined with paclitaxel, vinorelbine, gemcitabine and topotecan, respectively [5]. Despite these encouraging results, however, most of the responses are partial and of short duration.

In gynecologic cancers, we have always used conventional EBRT, which uses bony landmarks to define the target volume for pelvic radiotherapy. Treatment is delivered either with anterior and posterior opposed fields or with a 4-field box technique. There is a risk of a “marginal miss” using conventional EBRT if tumor falls outside of traditional field borders established by bony landmarks.

In recent years, development of three-dimensional radiation therapy (3D-RT) and intensity-modulated radiotherapy (IMRT) has increased the potential for an improved outcome in cervical cancer; however, there are few published reports on recurrent and metastatic cervical cancer. In comparison to conventional EBRT, 3D-RT and IMRT allow a more precise dose distribution conforming to the target volume and have less normal tissue morbidity. In particular, IMRT can achieve highly conformal dose distribution around the target with a steep dose gradient outside the targets, thus sparing OARs and providing an opportunity for dose escalation. This is particularly important for the treatment of recurrent and metastatic disease, especially in patients with a history of irradiation.

**Figure 1:**
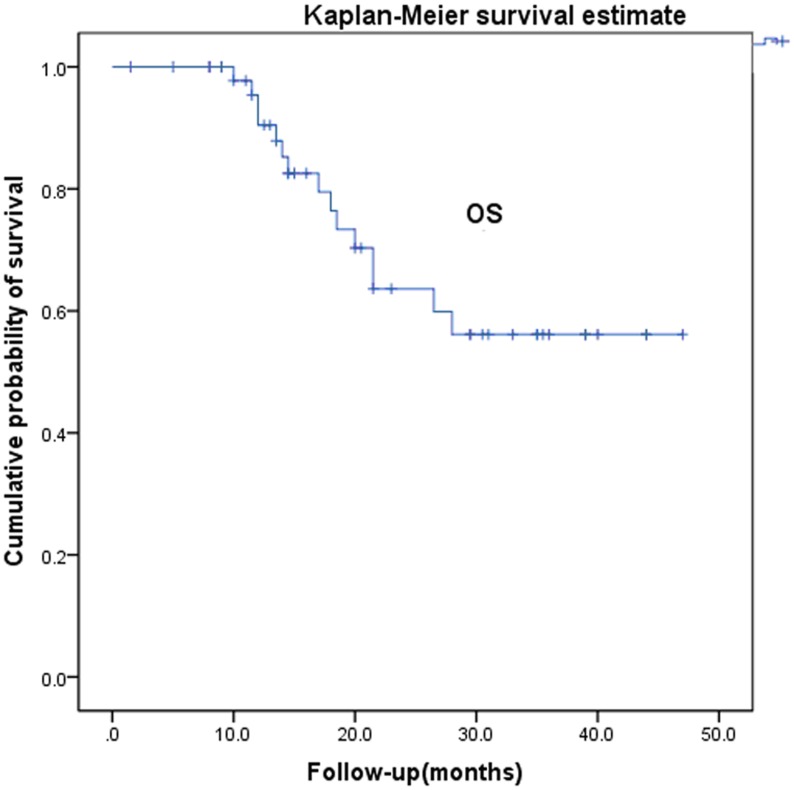
Overall survival after radiotherapy for recurrent and metastatic cervical cancer.

## Results

### Treatment

Seven patients who had received a prior irradiation treatment showed out-field relapse (n = 4) or PALN metastasis (n = 3). Thirty-nine patients showed bulky recurrence (≥3 cm). Eight patients, including 3 patients who received concomitant IMRT treatment for the recurrent tumor inside the pelvic region, were treated with PALN-IMRT with a median dose of 50 Gy. Direct tumor boost was planned for 13 patients. The prescription dose for PTV1 was 45 Gy, and the median dose of PTV2 was 57 Gy (range, 55–60 Gy). The median dose for PTV in 37 patients without direct tumor boost was 50 Gy (range, 45–64 Gy). All the patients completed the treatment.

**Figure 2:**
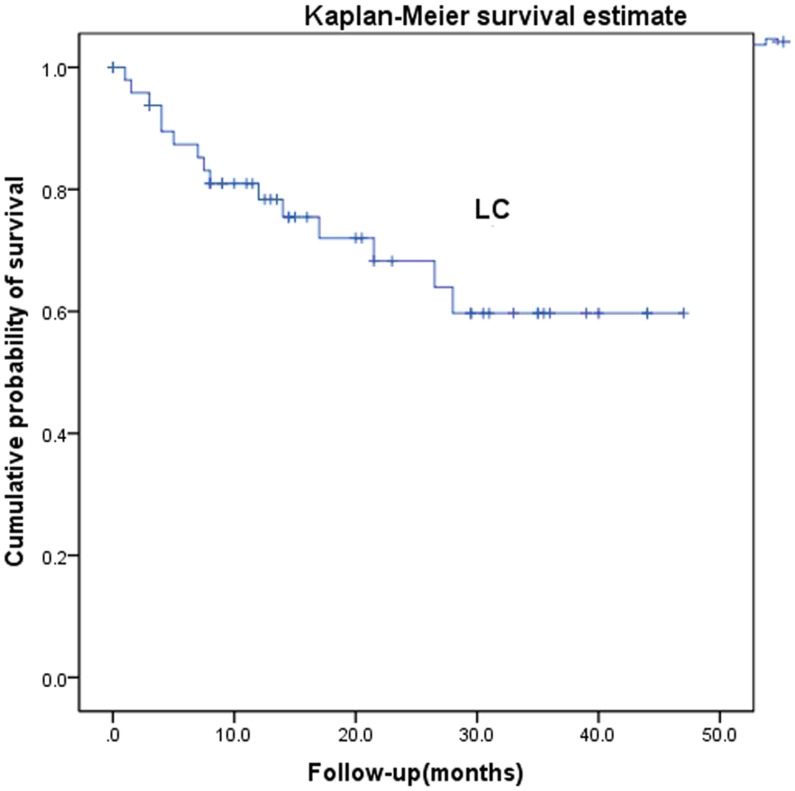
Local control after radiotherapy for recurrent and metastatic cervical cancer.

**Figure 3:**
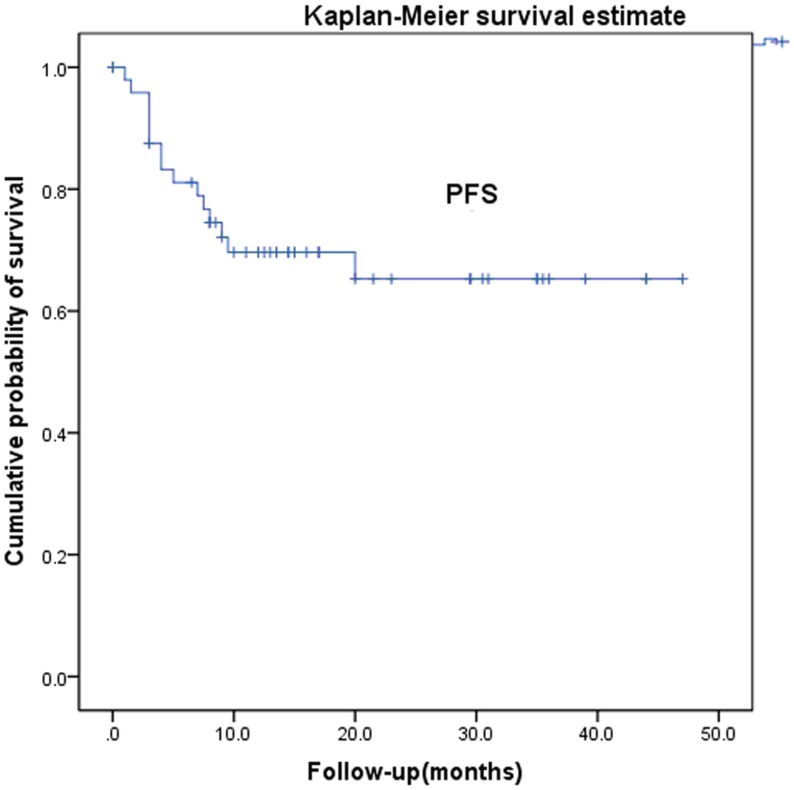
Progression-free survival after radiotherapy for recurrent and metastatic cervical cancer.

**Figure 4:**
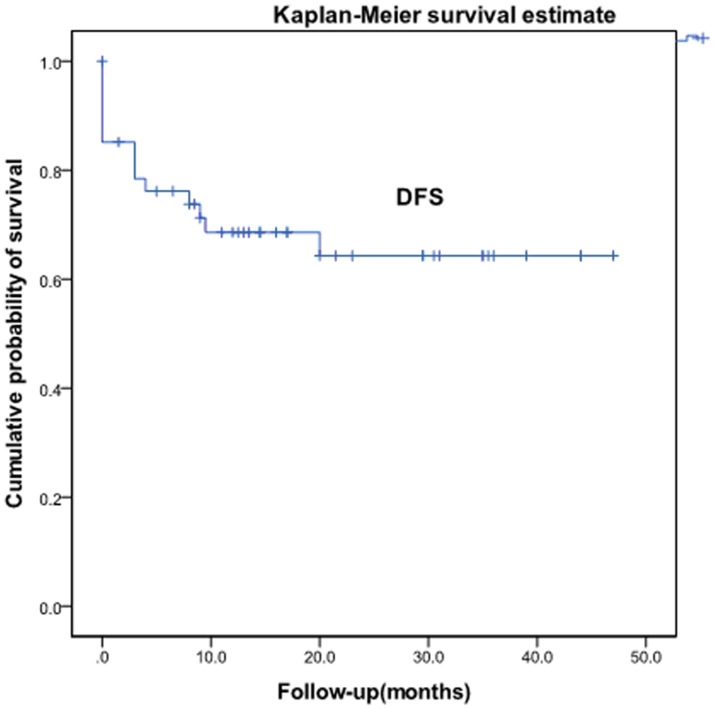
Disease-free survival after radiotherapy for recurrent and metastatic cervical cancer.

**Table 1:** Cox proportional hazard model analysis of variables predicting the overall survival.

	P	RR	95.0% CI
Age	0.533	1.028	0.942∼1.122
Time interval	0.039	0.917	0.845∼0.996
Salvage surger	0.118	0.155	0.015∼1.601
Concurrent chemotherapy	0.554	0.671	0.180∼2.508
Tumor size	0.183	1.187	0.922∼1.528
Histology	0.012	0.162	0.039∼0.669

### Survival, Systemic, and Locoregional Control

Two patients were lost to follow-up. Median follow-up period for all the patients was 18.3 months, with a minimum of 8 months for living patients. The 1-, 2-, and 3-year overall survival rates were 90.5%, 63.6%, and 56.1%, respectively (Fig. 1). The patient with the longest follow-up is still disease free at >47 months after the radiotherapy, with a 5-cm vaginal apex failure. The causes of death were systemic progression of disease (n = 13) and local tumor progression (n = 2). The 1-, 2-, and 3-year local control rates and progression-free survival were 78.4%, 68.2%, and 59.7% and 69.6%, 65.3%, and 65.3%, respectively (Figs. 2 and 3). The 1-, 2-, 3-year disease-free survival rates were 68.6%, 64.3%, and 64.3%, respectively (Fig. 4). Cox proportional hazard model analysis showed that the time interval to recurrence and histology were independent prognostic factors for overall survival (P<0.05) (Table 1). Overall survival was worse when the time interval between initial treatment and recurrence was less than 2 years (p = 0.013) and with non-squamous carcinoma (p = 0.012).

**Table 2:** Grade 3 acute toxicity during and up to 6 months after radiotherapy, and late treatment-related toxicities more than 6 months after the radiotherapy.

		No chemotherapy	With chemotherapy
Acute toxicity			
	Leukopenia	3	6
Late toxicity			
	Proctitis		1
	Intestinal obstruction		1

### Toxicity

Grade 5 acute toxicity was not observed. Nine patients developed grade 3 leukopenia and recovered quickly after the administration of G-CSF (granulocyte-colony stimulating factor). No patient developed grade 3 or greater acute gastrointestinal (GI) toxicity or genitourinary toxicity.

As for late toxicity, 7 patients developed Grade 2 proctitis, 1 patient had Grade 3 proctitis requiring surgical intervention, and 1 patient had Grade 3 intestinal obstruction and was treated with conventionally fractionated 60 Gy to the enlarged para-aortic lymph nodes. Grade 2 hematuria was observed in 3 patients (Table 2).

## Discussion

Although reports on 3D-RT or IMRT have been increasingly published in recent years, majority of studies have focused on locally advanced cervical cancer or whole pelvic radiotherapy after radical surgery, while few have addressed recurrent and metastatic disease. According to a retrospective review of more than 526 patients with invasive cervical cancer, 31% developed tumor recurrence, of which 58% recurred within 1 year and 76% within 2 years [6]. In the present study, the 1-year recurrence rate of 54% was in agreement with the previous reports, but only 32% patients showed recurrence within 2 years. The reported survival rates in patients with local recurrence of cervical cancer after a radical surgery range from 6% to 77%; moreover, patients with central recurrences had better prognoses than those with pelvic side wall recurrence [7], [8], [9], [10]. Median survival after diagnosis of recurrence was reported to be 9.8∼15 months [11], [12]. Tumor size was also an important prognostic factor, and more than 5-year survival was not observed in patients with tumor size of ≥3 cm [10]. The present study included 39 patients with bulky recurrence. The 3-year overall survival and locoregional control were 56.1% and 59.7%, respectively. Based on the benefits of 3D-RT or IMRT mentioned above, it is yet to be determined whether 5-year survival would be similar or even superior to the 3-year survival assessed in this study.

A study by Piver et al. reported a 5-year survival rate of only 9.6%, with death rates of 16.1% from complications and 74.1% from cancer. The intestinal complication rate in patients who received 6,000 cGy of split-course irradiation in 8 weeks was 61.9% and only 10.0% in the patients who primarily received 4,400–5,000 cGy in 4.5–5 weeks [13]. The result of RTOG 92-10 showed that twice-daily fractionation of PALN irradiation combined with CTX is highly toxic, resulting in an unacceptably high rate (17%, 5 of 29) of Grade 4 late toxicity. One patient died of acute complications of the therapy [14]. However, in comparison to PALN conventional radiation techniques, PALN-IMRT is feasible because of significant sparing of the critical normal structures [15], [16]. The radiation dose to the GTV was escalated from the conventional 45–60 Gy, whereas the PTV region received 45 Gy [17]. Eight patients in our study were treated with PALN-IMRT, with a median dose to PTV of 50 Gy. Of these, 3 patients developed grade 3 leukopenia and 1 patient developed grade 3 intestinal obstruction 10 months after the radiotherapy.

Stereotactic body radiotherapy (SBRT) is another treatment planning which results in high target dose and steep dose gradients beyond the target, and therefore, SBRT can deliver higher doses to tumors and causes less normal tissue damage. Recent years, there is a growing evidence of using stereotactic body radiotherapy for recurrent cervical cancers. Deodato et al. reported a case series of SBRT in recurrent gynecological cancer. 11 patients (12 lesions) were given a dose of 30 Gy in five fractions. After a median follow-up of 19 months, 7 patients (63%) experienced local and/or distant progression of disease. The 2-year local progression-free survival was 81.8%, while the 2-year metastases-free survival was 54.4%. Acute and late toxicities were grade 2 or less [18]. Due to large size of the recurrent cancer (median 4.5 cm) and peripheral location (n = 12), SBRT (median 3 fractions of 5 Gy to 65%) was used for local dose escalation after 50 Gy conventionally fractionated radiotherapy within all 19 patients (cervical cancer n = 12, endometrial cancer n = 7) in Guckenberger et al.’s report. 3-year overall survival was 34% with systemic progression the leading cause of death (7/10) after median follow-up of 22 months. 3-year local control rate was 81%. The rate of late toxicity > grade II was 25% at 3 years [19]. Despite the growing interest, there is very limited clinical data in the literature on SBRT for recurrent cervical cancer. Most reports have small sample sizes. No definite “optimal” SBRT single fraction dose and total dose have been achieved. Further studies on dose–response relationship are needed to evaluate the effectiveness and toxicity in recurrent cervical cancer.

In this retrospective study, we mainly used platinum-based chemotherapy. In 1999, the US National Cancer Institute recommended using cisplatin-based chemotherapy during radiation for cervical cancer based on several cooperative clinical trials that demonstrated the benefit of the concurrent use of chemotherapy and radiation to treat locally advanced and high-risk cervical cancer [20], [21], [22], [23], [24]. Nedaplatin, a second-generation platinum complex, is considered to have equivalent or more pronounced activity against solid tumors but less nephrotoxicity and gastrointestinal toxicity than cisplatin and, therefore, could be used as a substitute for cisplatin in the treatment of cervical cancer [25], [26]. The addition, concurrent paclitaxel administration, which targets different molecules as compared to platinum, may have an additive or even a synergistic anticancer effect [27]. In a phase II study, we reported that the use of paclitaxel (35 mg/m^2^) and nedaplatin (20 mg/m^2^) for concurrent chemoradiotherapy CCRT was effective and well tolerated [28]. In our study, concurrent platinum-based chemotherapy, with cisplatin/nedaplatin or cisplatin plus paclitaxel as the most common compounds, was administered in 38 patients. However, platinum-based chemotherapy did not significantly affect the rates of locoregional control and overall survival (p = 0.357 and p = 0.554, respectively). Low statistical power associated with small sample size may be a contributing factor. In our analysis of other predictive factors, the time interval of recurrence and histology were found to be statistically significant factors. However, the role of age, salvage surgery, and tumor size has not yet been well established.

Multiple studies on 3D-RT or IMRT have been performed in patients with cervical cancer that showed the clinical benefits of 3D-RT or IMRT, such as reduction in acute gastrointestinal and hematologic toxicity, over the conventional EBRT. Yamazaki et al. reported that bowel complications reduced from 17.5% to 2.9% and leg edema from 28.6% to 3.1% after using 3D-RT during the postoperative radiotherapy for cervical cancer as compared to parallel-opposed fields [29]. Grade 2 acute gastrointestinal toxicity was 60% vs. 91% (p = 0.002) in Mundt et al.’s retrospective study that compared 40 gynecology patients who underwent IMRT of which 35 were previously treated with conventional EBRT. Grade 3 toxicity did not develop in any of the patients. No or only infrequent antidiarrheal medications were needed (75% vs. 34%, p = 0.001). Grade 2 genitourinary morbidity was reduced from 20% to 10% after administration of IMRT [30], and chronic GI toxicity was 11.1% vs. 50.0%, (p = 0.001) [31]. In Roeske’s analysis, the most significant factor that was correlated with acute GI toxicity was the volume of small bowel receiving the prescription dose of 45 Gy [32]. IMRT is also a good means of reducing hematological toxicity, because 40% of the total body bone marrow reserve lies within the pelvic bones. Meanwhile, concurrent chemoradiotherapy has become a standard treatment for locally advanced cervical cancer. Moreover, the use of concurrent chemotherapy increases the likelihood of developing clinical myelotoxicity. Patients using intensity-modulated whole pelvic radiotherapy experienced lesser Grade 2 or greater WBC toxicity than conventional whole pelvic radiotherapy (31.2% vs. 60%, p = 0.08) [33], which provides the opportunity for additional use of novel systemic therapies. In our study, 9 (18%) patients developed grade 3 leukopenia, which occurred less frequently and showed a faster recovery after the administration of G-CSF. Nine (18%) patients developed Grade 2 or higher late GI toxicity, including 1 patient who developed Grade 3 proctitis requiring surgical intervention and 1 patient who developed Grade 3 intestinal obstruction. These rates are slightly higher than those reported previously [31] but appeared to be acceptable when the median dose prescribed for the PTV is considered. Based on the rather short follow-up of our patients, the true incidence of late toxicity may be underestimated. Therefore, long-term follow-up studies are needed to confirm these results.

3D-RT or IMRT for recurrent and metastatic cervical cancer is an effective treatment method with a 3-year overall survival rate of 56.1% and an acceptable level of complications. Longer follow-up and further studies are needed to confirm the role of 3D-RT or IMRT in the multimodality management of the disease.

## Materials and Methods

All of the procedures were done in accordance with the Declaration of Helsinki and relevant policies in China. The study obtained ethics approval for my study from the ethics committee of Shanghai Cancer Hospital of Fudan University. We obtained the informed consent from all participants involved in our study.

### Patient Characteristics

Between March 2007 and December 2010, 50 patients with recurrent cervical and metastatic cancer were treated with 3D-RT or IMRT. The patient characteristics are summarized in Table 3.

The median interval to recurrence or metastases was 12 months (range, 6–51 months). Because of difficulties in obtaining a histological diagnosis, all the patients underwent physical examination, computed tomography (CT), and/or magnetic resonance imaging (MRI) of the chest, abdomen, and pelvic cavity before the treatment. Certain patients underwent positron emission tomography (PET) for a general evaluation. Surgery was performed before irradiation in 5 patients (10%). Sixteen patients (32%) underwent 3D-RT, and 34 patients (68%) received IMRT.

**Table 3:** Patient characteristics.

Characteristics		N = 50	%
Age (years)			
	Average	43	
	Range	27–73	
Histology			
	Squamous cell carcinoma	43	86
	Adenocarcinoma	2	4
	Adenosquamous cell carcinoma	4	8
	Mucinous adenocarcinoma	1	2
Prior treatment			
	Surgery	31	62
	Surgery and adjuvant chemotherapy	12	24
	Surgery and adjuvant radiotherapy	4	8
	Surgery and adjuvant chemoradiotherapy	3	6
Site of recurrence or metastases			
	Pelvic wall recurrence	23	46
	Central recurrence	17	34
	PALN metastases	5	10
	Pelvic wall recurrence and PALNmetastases	2	4
	Central recurrence and PALN metastases	1	2
	Central recurrence and ILN metastases	2	4
Size of the pelvic recurrence			
	<3 cm	6	12
	> = 3 cm	39	78

Abbreviations: PALN: Para-aortic lymph nodes; ILN: Inguinal lymph nodes.

Induction chemotherapy was administered in 3 patients (nedaplatin and paclitaxel, n = 2; nedaplatin and cyclophosphamide, n = 1), and concurrent platinum-based chemotherapy was administered in 38 patients (76%) (weekly cisplatin (30 mg/m^2^), n = 9; weekly cisplatin and paclitaxel (30 mg/m^2^), n = 15; weekly nedaplatin (20 mg/m^2^), n = 7; 3-weekly cisplatin (80 mg/m^2^) and paclitaxel (135 mg/m^2^), n = 7).

### Target Definition

Contrast-enhanced planning CT of the pelvic or abdominal region was performed in the treatment position. Targets and organs at risk (OARs) were delineated on axial CT slices. A 7-mm isotropic expansion of the gross tumor volume (GTV) encompassing the tumor and/or pathological lymph nodes gave the clinical target volume (CTV). In patients who had undergone a surgery, the CTV encompassed the postoperative tumor bed. The CTV was expanded isotropically with an 8 -mm margin to yield a planning target volume (PTV). The spinal cord, kidney, small bowel, bladder, rectum, and caput femoris were outlined as the OARs.

Direct tumor boost is an attractive method for the treatment of recurrent and metastatic cervical cancer that allows the maintenance of standard doses and fractionation to the areas of potential microscopic spread while improving the therapeutic ratio by delivering a higher total dose to all of the macroscopic disease. Patients can be treated with IMRT for the initial treatment volume (PTV1) followed by a direct IMRT booster dose delivered to a smaller volume (PTV2).

### Dose Prescription, Treatment Planning, and Delivery

For the PTV encompassing the macroscopic tumor, a median dose of 60 Gy in 30 fractions and 45 or 50 Gy in 25 fractions was prescribed for pelvic recurrence and para-aortic lymph nodes PALN metastasis, respectively. The prescribed dose for direct tumor boost was 12 or 14 Gy with a single dose of 2 Gy for PTV2 after 45 or 50 Gy in 25 fractions given for PTV1. This dosing strategy aimed to deliver at least 95% of the prescribed dose to at least 95% of the PTV and not more than 105% of the prescribed dose to not more than 1% of the PTV. A maximum cumulative dose constraint of 40 Gy was implemented for the spinal cord. Treatment was delivered using 6-MV photon beams from a linear accelerator.

### Follow-up and Statistical Analysis

During radiotherapy, all the patients were followed up at least once weekly. Acute toxicity was assessed according to the Common Toxicity Criteria (CTC) version 2.0; for late radiotherapy toxicity, the RTOG (Radiation Therapy Oncology Group) criteria were used.

The endpoint for time-to-event analysis was the overall survival (death from any cause), which was calculated using the Kaplan–Meier method. Local tumor control was defined as tumor shrinkage and no tumor progression during follow-up. Determination of local control required CT or MRI imaging and gynecological examination. Progression-free survival (freedom from disease progression) and disease-free survival (free of residual disease) were also calculated. All the events were measured from the first day of radiotherapy to the date of tumor occurrence or the last follow-up for the censored observations. All the analyses were performed using SPSS 15.0 software.
