# Correction: 3D Radiation Therapy or Intensity-Modulated Radiotherapy for Recurrent and Metastatic Cervical Cancer: The Shanghai Cancer Hospital Experience

**DOI:** 10.1371/annotation/1d6063be-ff28-4a65-a3a0-bcaf076eab4b

**Published:** 2012-09-27

**Authors:** Su-Ping Liu, Xiao Huang, Gui-Hao Ke, Xiao-Wei Huang

There is an error in the authors' affiliation. The correct affiliation is: Department of Gynecologic Oncology, Fudan University Shanghai Cancer Center; Department of Oncology, Shanghai Medical College, Fudan University, Shanghai, China

